# Environmental and Contextual Considerations

**Published:** 2004

**Keywords:** adolescent, underage drinking, environmental factors, risk factors, protective factors, drinking and driving, alcoholic beverage, AOD (alcohol and other drug) product advertising, AOD price, sales and excise tax, minimum drinking age laws, zero tolerance laws

## Abstract

A number of environmental factors can influence an adolescent’s risk for drinking, including parenting styles, an adolescent’s choice of peer groups, and even whether he or she is active in after-school activities. Alcohol advertising, the price of alcohol, and the degree to which underage drinking laws are enforced also play a role. It is difficult to establish the degree to which alcohol use is influenced by environmental factors. This article describes some of the environmental influences that may increase the risk for underage drinking.

## Overview

The spectrum of environmental factors that can influence an adolescent’s drinking ranges from parents and family to the community at large and includes the availability, price, and advertising of alcohol. For a variety of reasons, measuring the impact of an environmental feature on drinking in a young person can be a challenge. Research has found, for example, that adolescents with supportive parents who monitor their children’s activities are less likely to be involved in risky behaviors than adolescents with less attentive parents. At the same time, genetic influences on personality can influence parenting styles as well as choice of peer groups and involvement in activities. Innate traits may help prompt an adolescent to, for example, choose a peer group inclined to risky behavior; however, that peer group is itself an environmental factor that encourages risky activity. One goal of research is to be able to provide an understanding of the interactions of genetics vs. environmental factors and their relative contributions to risky behavior.

On a larger scale, alcohol advertising is pervasive in this culture, and much of it is presented in ways that appeal to youth. Some research suggests an association between adolescents’ reactions to alcohol advertising and their desire or intention to drink. Results have been mixed, however, in studies aimed at establishing whether alcohol advertising actually causes youth to drink.

In contrast, most studies looking at the impact of alcohol price or tax changes on youth have found that young people’s alcohol consumption drops significantly in response to tax and price increases. Other research has examined the effects of alcohol prices or taxes on the harmful consequences of drinking; most studies looking at traffic fatalities have found that higher prices and taxes are associated with reductions in traffic crash fatalities among younger drivers.

All States now have laws making it illegal to sell alcohol to people younger than age 21. Numerous studies have established the effectiveness of underage drinking laws in reducing both drinking and alcohol-related crashes among people under age 21. The National Highway Traffic Safety Administration (NHTSA) estimates that a legal drinking age of 21 saves 700 to 1,000 lives annually. All 50 States now also have zero-tolerance laws, which make it illegal for people younger than age 21 to drive after any drinking. These laws also have contributed to declines in alcohol-related traffic deaths among those younger than age 21. For a number of reasons, zero-tolerance laws have not been vigorously enforced. This lack of vigorous enforcement occurs in spite of evidence from studies done before universal adoption of zero-tolerance laws indicating that States instituting these laws saw substantial declines in the proportion of people younger than age 21 who drove after any drinking.

## Parents, Peers, and Community Influences

Parenting styles, choice of peer group, and the community context in which adolescents are raised have all been heavily researched as possible risk-promoting or protective influences on drinking-related outcomes (Halpern-Felsher and Biehl 2004). As might be anticipated, numerous studies have found that children with loving, supportive, and involved parents had better developmental outcomes and were less likely to use alcohol than children raised in less supportive homes. Parental support encompassed monitoring their children’s activities while supporting their independence and setting limits (Barnes et al. 2000; Bogenschneider et al. 1998; Reifman et al. 1998; DiClemente et al. 2001; Davies et al. 2001; Steinberg et al. 1994).

However, although parents’ awareness of their children’s activities is certainly important, as is controlling their whereabouts, the source of parents’ information about what their children are doing also is critical. Research suggests that facilitating children’s willingness to share information about their lives may be associated with better outcomes (Stattin and Kerr 2000).

Parents who drank more and who held favorable views about drinking had offspring who drank more. Similarly, adolescents who spent more time with peers who consumed alcohol were more likely to drink (Colder and Chassin 1999; Curran et al. 1997; Sieving et al. 2000; Stice et al. 1998).

Although it is tempting to conclude that the variables noted above truly have a causal influence on drinking, the evidence at this point does not establish a causal or mediational influence. Because we know that genetic risk for alcohol use patterns can manifest itself through personality variables, and that these same personality variables can influence parenting styles, choice of peer groups, and even engagement in after-school programs (and other environmental choices), it is difficult to establish the degree to which alcohol use is influenced independently by parents, peers, and environment. These questions must be pursued using genetically informed research designs and by experiments in which these variables are manipulated independently of genetic factors.

## The Role of Advertising

Youth are exposed to a significant amount of alcohol advertising. Alcohol ads appear in virtually all types of media. Such ads are common on television and often are presented in ways that appeal to youth and are shown at times when many youth are likely to see them. Half of televised beer ads, for example, air during Saturday or Sunday afternoon sporting events—programs that are popular among youth (Snyder et al. 2000). Beer is the beverage of choice for many youth, and between 1998 and 2002, industry spending on televised beer ads increased 45 percent to $972 million. Over the same period, spending on liquor advertising increased 530 percent to $18 million (Center for Science in the Public Interest [CSPI] 2003). Youth also routinely see ads for alcoholic beverages in magazines, on billboards, and on the Internet. For example, the Center on Alcohol Marketing and Youth (CAMY) found that youth saw 49 percent more beer ads and 20 percent more distilled spirits ads than did adults (CAMY 2004). CAMY researchers also reported that 12- to 17-year-olds hear more alcohol ads on the radio than do adults (CAMY 2003). Radio alcohol ads were frequently placed on stations with youth formats and were aired when youth were most likely to be listening (CAMY 2003). A study of Internet use by youth found that alcohol-related Web sites contained features appealing to youth, such as video games and cartoons, but had few effective mechanisms to keep underage youth from accessing the Web sites (CAMY 2004).

Scientists are trying to determine how advertising affects youth generally and underage alcohol consumption more specifically. A simple model of the effects of alcohol advertising would posit that greater amounts of advertising lead to more exposure to advertising, which leads to more drinking. Thus, much of the research in this area has been focused on explicating part or all of this sequence by: quantifying the number of alcohol portrayals in various media (including advertising); estimating exposure to advertising in various populations; studying whether exposed populations recall and are aware of alcohol advertising; examining how awareness affects alcohol expectancies and intention to drink; studying cross-sectionally the association between advertising and alcohol outcomes; and studying prospectively the causal relationships among advertising variables and drinking outcomes, such as initiation, escalation, and levels and frequency of consumption. Among these, the longitudinal studies are of greatest interest because they have the potential to address the fundamental questions of cause and effect.

Assessing the effect of advertisements on the drinking behavior of individuals or populations is a complicated endeavor. It often is difficult to ascertain the specific effects of advertising because they must be measured against a background dense in alcohol messages and images. In addition, advertisements or alcohol-related messages will influence different individuals and different populations differently at different developmental stages and times in their lives. And furthermore, the mechanisms by which advertising may affect actual drinking behavior have not been extensively studied and are not well understood.

One line of research in this area has directly studied young people’s reactions to alcohol advertisements and the correlates of those reactions. A study of third, sixth, and ninth graders showed that the third grade children who found alcohol ads desirable also were more likely to see positive benefits from drinking and to desire products with alcohol logos. Older children in the study who found the ads and logo products appealing were more likely to already be engaged in drinking behaviors (Austin and Knaus 2000). A related survey of 9th and 12th grade students examined the effect of media exposure on drinking behavior (Austin et al. 2000). Students reported on their television viewing habits, viewing perceptions, desire for alcohol products, and alcohol use. Findings supported a positive and indirect effect of media on adolescent drinking. The media influence beliefs about the appeal and desirability of alcohol, and the beliefs in turn influence drinking (Austin et al. 2000).

Another study examined brain response to viewing alcoholic beverage pictures and nonalcoholic beverage pictures in 15- to 17-year-old heavy drinkers and nondrinkers using functional magnetic resonance imaging. Heavy-drinking teens showed substantially greater brain activation while viewing the alcohol ads relative to the nonalcohol ads, and this pattern differed significantly from that of nondrinkers (Tapert et al. 2003). Brain regions showing differential brain response suggested that heavy-drinking teens attended more closely, recalled pleasure and positive affect, and generated increased appetitive response while viewing an assortment of alcohol advertisements. On the other hand, Zogg and colleagues (2004), in an expectancy study of perceived positive and negative outcomes of alcohol use, found no predictive effects of exposure to televised alcohol advertisements, televised sports (which is dense in alcohol advertising), or firsthand observation of others drinking.

**Social Policy and Law**

In a society in which alcohol is widely available and aggressively promoted and where alcohol use remains normative behavior among youth, what social policies should be adopted toward adolescent drinking? This question is not just about defining the legal drinking age. Social policy and law are not the same thing. Law is one tool of social policy, with some advantages (e.g., deterrence) and some disadvantages (e.g., individual and social costs of enforcement). Institutions of social control offer another means of strongly discouraging alcohol use by youngsters under a certain age, regardless of what age the law defines as the minimum for legal alcohol consumption. An important goal of research is to determine the ways in which the law can most efficiently be deployed while encouraging nonlegal institutions to play a more substantial role than they now do.

***Age of Lawful Access***

The law can use different ways of drawing the line between legal and illegal conduct. Right now, the law, by and large, uses an approach to defining the legal age of access to alcohol that is both binary (legal or not) and categorical (based on a simple age classification). An example of another type of approach is graduated licensing of young drivers, in which conditions are placed on driving during a transitional phase before they have unrestricted access to driving a car.

Research is needed to explore the role of alcohol use in the lengthening transition from adolescence to adulthood, including what some investigators have called the periods of emerging adulthood and young adulthood. This research should be linked to studies of other developmental domains, including work and relationships with sexual partners and parents. This body of research may prove to have important implications for all transitional legal arrangements.

***Sanctions for Underage Drinkers***

An important objective with regard to drinking laws and youth is identifying the appropriate sanctions for violators. The policy challenge is to optimize the usefulness of laws against underage possession and related offenses. This requires attention to the types of sanctions that are needed as well as the enforcement strategies and judicial procedures that are used. In general, the goal should be to increase the declarative and deterrent effects of the law without harming the young person’s future life prospects. These judgments require research on a variety of issues, including the attitudinal and behavioral effects of different types of sanctions, different types of adjudicatory procedures, and different types and levels of enforcement. More generally, a better understanding is needed of the attitudes of young people at different developmental stages toward obedience to law, and of the ways in which decisions regarding use of alcohol (as well as tobacco, marijuana, and other drugs) affect and are affected by attitudes toward the law. These inquiries need to be tied to the developmental perspectives used to understand other aspects of underage alcohol consumption*.*

***Ways of Raising the “Price” of Underage Drinking***

Threatening to punish young people for obtaining alcohol is one way of raising the price of alcohol use. Another is to curtail the supply by deterring retailers and other adults from selling or giving alcohol to underage drinkers. Curtailing the supply makes the young person spend more time looking for alcohol, thereby increasing the “search costs.” Restricting outlets also can do this. A final way to raise the price is by increasing excise taxes. One political concern raised by tax increases and outlet restriction is that (unlike the other mechanisms) these tools also raise the price for adult purchasers. All of these issues require systematic understanding of where and how underage drinkers get their alcohol and, more generally, of the market for youthful drinking.

Image advertising, which focuses on the lifestyle of the product user rather than the product itself, is preferred by underage youth (seventh grade) and has been associated with intentions to drink in the future (Kelly and Edwards 1998). A study involving male and female Anglo and Latino adolescents found that, both for males and females, positive responses to beer advertisements were associated with greater present and planned alcohol use. No differences were found related to ethnicity (Slater et al. 1997). Another study conducted focus group discussions with students ages 9 to 15 to learn what aspects of television alcohol advertisements made them attractive to young people. The students responded positively to ads with humor, talking animals, and youthful lifestyle appeal and negatively to the product focus of the ads (Waiters et al. 2001).

Although they are informative and interesting, these studies do not address the question of causality: Do alcohol advertisements cause youth to drink, or do youth who already drink pay more attention to alcohol advertising?

Two recent cross-sectional studies found positive associations between advertising and consumption. Collins and colleagues (2003) measured advertisement awareness, drinking beliefs, and drinking behavior among eighth grade students. These researchers found that boys are more likely to be aware of and remember beer marketing and may be more likely to drink as a result of this awareness.

Another study examined whether recall of and liking of alcohol advertisements leads to greater intentions to drink in the future and higher consumption of alcohol (Chen and Grube 2001). This study sampled students in grades 5 to 8 and grades 9 to 11 and measured their response to 16 alcohol ads and 4 soft drink ads. The study found that liking specific elements of alcohol ads (characters, humor, story line) predicted liking the advertisements, and that liking the advertising directly predicted current drinking levels and had significant indirect effects on drinking and future intentions to drink. Results of earlier studies that examined the relationship between liking alcohol advertising and current and future intentions to drink, however, were mixed (Kelly and Edwards 1998; Wylie et al. 1998).

A few prospective studies also have addressed this issue. A longitudinal study of New Zealand youth found that liking alcohol advertising at age 18 was related to higher levels of beer consumption at age 21 (Casswell and Zhang 1998). Two additional recent prospective studies found a positive relationship between exposure to advertising and consumption. Ellickson and colleagues (2003) found in a sample of seventh grade drinkers and nondrinkers from North Dakota that several forms of advertising predicted future adolescent drinking for both groups. And Stacy and colleagues (2004) found that exposure to advertising increased the risk of subsequent beer consumption.

Another group of potentially informative investigations are econometric studies of the relationship between alcohol advertising and consumption. Results of these studies also have been mixed. A study by Saffer (2002) found that advertising increased consumption, whereas other studies found that alcohol advertising affects brand choice but not overall consumption (Nelson and Moran 1995; Gius 1996). Another study by Saffer and Dhaval (2003) suggests that a complete ban on alcohol advertising might reduce the prevalence of monthly drinking by 12- to 18-year-olds from about 25 percent to 21 percent and of binge drinking from 12 percent to 7 percent (Saffer and Dhaval 2003). Despite their potential effectiveness for reducing underage drinking, comprehensive advertising bans are not likely to receive public support, and partial bans are likely to prompt the alcohol industry to increase their ads in other media (Saffer 2002).

**By the Numbers: Alcohol Advertising, Price, and Legislation**

- Industry spending on TV beer ads, 2002: $972 million (up 45 percent from 1998) (CSPI 2003).
- Industry spending on TV liquor ads, 2002: $18 million (up 530 percent from 1998) (CSPI 2003).
- Young people see 49 percent more beer ads and 27 percent more ads for distilled spirits than adults see (CAMY 2004).
- Studies have found that young people’s alcohol consumption drops significantly in response to price or tax changes, in some cases exceeding the reductions estimated for the general population (Grossman et al. 1987; Coate and Grossman 1988; Kenkel 1993; Sutton and Godfrey 1995; Ruhm 1996; Grossman et al. 1998).
- When States increased the legal drinking age to 21, alcohol-related crashes among people younger than 21 decreased an average of 16 percent (Shults et al. 2001).
- NHTSA estimates that the legal drinking age of 21 saves 700 to 1,000 American lives annually, and has prevented more than 21,000 traffic deaths since 1976 (NHTSA 2003).
- The first 30 States to adopt zero-tolerance laws had a 19-percent decline in the proportion of people younger than 21 who drove after drinking, when compared with States without these laws, and a 23-percent decline in the proportion who drove after five or more drinks (Wagenaar et al. 2001).

In general, research on the impact of alcohol advertising on actual drinking behavior has been mixed, and observed effects have been small. In addition, many of the cited studies are subject to recall bias. Furthermore, many studies have been cross-sectional, making it difficult to draw definitive conclusions about the relationship between advertising and alcohol consumption (Grube 2004).

## The Effect of Price on Adolescent Alcohol Consumption

A substantial body of research has shown that higher prices or taxes on alcoholic beverages are associated with lower levels of alcohol consumption and alcohol-related problems (Leung and Phelps 1993; Kenkel and Manning 1996; Chaloupka et al. 1998; Cook and Moore 2002). Estimates vary, however, in the extent to which consumption or problems change in response to a given price or tax change. Some studies have examined these effects among young people separately from the general population. Most such studies have found that young people’s alcohol consumption drops significantly in response to price or tax changes, in some cases exceeding the reductions estimated for the general population (Grossman et al. 1987; Coate and Grossman 1988; Kenkel 1993; Sutton and Godfrey 1995; Ruhm 1996; Grossman et al. 1998). An exception is the study by Dee (1999), which found only small and statistically insignificant effects of beer taxes on teens’ drinking behavior. In addition, Chaloupka and Wechsler (1996) found that, although higher beer prices tend to decrease drinking and binge drinking among U.S. college students, price is a relatively weak tool for influencing these behaviors, especially among males. In a study of the population age 17 and older, Manning and colleagues (1995) found that alcohol consumption decreased in response to price increases for all but the top 5 percent of drinkers, who exhibited no significant price response. Several studies have examined the effects of alcohol prices or taxes on traffic crash fatalities and other alcohol-related problems. Most such studies have reported that higher taxes or prices were associated with significant reductions in traffic crash fatalities or drunk driving, particularly among younger drivers and during nighttime hours (Saffer and Grossman 1987; Chaloupka et al. 1993; Kenkel 1993; Ruhm 1996). A few later studies have questioned these findings. Dee (1999) found some evidence that beer taxes tend to reduce teen traffic fatalities but concluded that those results were not robust and should be viewed with skepticism. Young and Likens (2000) found no significant effects of beer taxes on traffic crash fatality rates, either for young drivers or the general population. Mast and colleagues (1999) found mixed results, with several analyses indicating significant but relatively small effects of beer taxation on traffic fatalities. Other research has found associations between higher alcoholic beverage taxes and lower rates of some types of violent crime (Cook and Moore 1993*a*), reduced incidence of physical child abuse committed by women (Markowitz and Grossman 2000), and lower rates of sexually transmitted diseases (Chesson et al. 2000), as well as with increases in college graduation rates (Cook and Moore 1993*b*).

Further research is needed to clarify the effects that alcoholic beverage prices or taxes have on different drinking behaviors, health-related outcomes, and population subgroups, and to reconcile conflicting findings that have appeared in the literature. To date, however, the weight of evidence suggests that higher prices and taxes can help to reduce alcohol consumption and alcohol-related problems.

## The Effect of Drinking Laws on Alcohol Consumption by Adolescents

### Legal Drinking Age of 21

In 1984, when 25 States had a legal drinking age of 21, the U.S. Congress passed legislation that would withhold highway construction funds from States that did not make it illegal to sell alcohol to people younger than age 21. By 1988, all States adopted such a law. A review of more than 49 studies of legal drinking age changes revealed that in the 1970s and 1980s, when many States lowered the drinking age, alcohol-related traffic crashes increased 10 percent. In contrast, when States increased the legal drinking age to 21, alcohol-related crashes among people younger than age 21 decreased an average of 16 percent (Shults et al. 2001). Wagenaar and Toomey (2002) reviewed more than 48 studies of the effects of drinking age changes on drinking and 57 studies of traffic crashes. They concluded that increases in the age of legal alcohol purchase and consumption have been the most successful intervention to date in reducing drinking and alcohol-related crashes among people under age 21. One national study of laws raising the drinking age to 21 indicated that people who grew up in States with a drinking age of 21 relative to those with lower legal drinking ages drank less not only when they were younger than age 21 but also when they were ages 21 to 25 (O’Malley and Wagenaar 1991). NHTSA (2003) estimates that a legal drinking age of 21 saves 700 to 1,000 lives annually and that more than 21,000 traffic deaths have been prevented by such laws since 1976.

### Zero-Tolerance Laws

All States now have zero-tolerance laws that make it illegal for people under age 21 to drive after any drinking. These laws also have contributed to declines in alcohol-related traffic deaths among people younger than age 21. A comparison of the first eight States to adopt zero-tolerance laws with nearby States without such laws revealed a 21-percent greater decline in zero-tolerance law States in the proportion of fatal crashes among drivers younger than age 21 that were of the type most likely to involve alcohol (i.e., single-vehicle fatal crashes at night) (Hingson et al. 1994). Wagenaar and colleagues (2001) found that in the first 30 States to adopt zero-tolerance laws, relative to the rest of the nation, there was a 19-percent decline in the proportion of people younger than age 21 who drove after any drinking and a 23-percent decline in the proportion who drove after five or more drinks.

Unfortunately, despite their demonstrated benefits, legal drinking age and zero-tolerance laws generally have not been vigorously enforced (Jones and Lacey 2001). Young drivers are substantially underrepresented in the driving while intoxicated (DWI) arrest population relative to their contributions to the alcohol-crash problem (Preusser et al. 1992; Voas and Williams 1986). Younger drivers may be more likely to drink in locations where DWI enforcement resources are less likely to be deployed. Young drivers with high blood alcohol concentrations also are more likely to be missed by police at sobriety checkpoints (Wells et al. 1997).

Stepped-up enforcement of alcohol purchase laws aimed at sellers and buyers can be effective (Preusser et al.1994; Wagenaar et al. 2000) if resources are made available for this purpose. Enforcement of zero-tolerance laws is hindered in some States because their implied-consent laws require either an arrest for DWI or probable cause for a DWI arrest before the evidentiary test can be done to prove a zero-tolerance violation (Ferguson et al. 2000). Thus, in practice, zero-tolerance laws often are not enforced independently of DWI. In States such as New Mexico, where this situation exists, the majority of teenagers are unaware that there is a zero-tolerance law (Ferguson and Williams 2002).
